# In Silico Redesign of Epstein-Barr Virus Entry Glycoproteins to Redirect Cellular Tropism as a Foundation for a Novel B Cell Oncolytic Therapy

**DOI:** 10.7759/cureus.97744

**Published:** 2025-11-25

**Authors:** Jeevpreet Ahluwalia, Aarush Katakam, Sanjith Satish

**Affiliations:** 1 Medicine, The Innovative STEMagazine, College Station, USA

**Keywords:** b-cell biology, epstein–barr virus (ebv), novel anti-cancer, novel mutation, oncolytic viruses

## Abstract

B cell acute lymphoblastic leukemia (B-ALL) affects thousands of patients each year, with relapse occurring in some cases due to residual malignant B cells following chemotherapy or immunotherapy. Relapsed disease has a median survival of only a few months under conventional salvage therapy and a markedly elevated mortality rate.Oncolytic viruses offer an alternative treatment that works by specifically replicating within cancer cells, inducing cytolysis, and replicating and amplifying the viral particles at the site of infection until the tumor is eliminated, a mechanism valuable for eliminating residual disease that traditional therapies fail to eliminate. Epstein-Barr virus (EBV) is an ideal oncolytic candidate because of its natural tropism to B cells, where the virus enters through gp42 (glycoprotein 42, a viral protein) binding to HLA (human leukocyte antigen) class II molecules on B cells before entering the cell through gH/gL (glycoprotein H/glycoprotein L) viral glycoprotein complex on the viruses' outer membrane)-mediated membrane fusion. However, EBV can also infect epithelial cells through an alternative pathway in which the gH/gL complex binds to the EphA2 (ephrin type-A receptor 2) receptor before triggering membrane fusion, creating an off-target pathway with potential for toxicity, uncontrolled viral replication, and inflammatory response that prevents clinical translation. If the gH/gL-EphA2 interaction is disrupted, however, then this off-target concern would be addressed.

We therefore computationally reengineered the viral gH/gL complex to selectively disrupt the gH/gL-EphA2 interaction while avoiding disruption of the gH/gL/gp42 complex stability required for B cell entry. The residues to be mutated were chosen through quantitative analysis on PDBePISA; mutant complexes were modeled using AlphaFold-Multimer (DeepMind Technologies, London, UK), refined through HADDOCK docking (Bonvin Lab, Utrecht University, Netherlands), and validated through 20-ns molecular dynamics simulations in OpenMM (Simbios Center, Stanford University, CA, USA). Analysis of the complexes showed success in selective disruption: the mutant gp42-HLA class II complex showed stability with a root mean square deviation (RMSD) of 2.29 ± 0.18 Å and mean root mean square fluctuation (RMSF) of 1.31 Å when compared to the mutant gH/gL-EphA2 interaction, which showed significant instability with an RMSD of 4.77 ± 0.22 Å and mean RMSF of 2.11 Å, with 46% of residues exceeding the 2.0 Å flexibility.

In summation, this paper intends to lay an initial computational framework for a B cell-selective oncolytic EBV variant that eliminates epithelial off-target effects while maintaining therapeutic efficacy against leukemic B cells.

## Introduction

B cell acute lymphoblastic leukemia (B-ALL) is the most prevalent childhood acute lymphoblastic leukemia and a leading cause of pediatric cancer mortality [1]. Although chemotherapy achieves remission in most patients, 10-20% face a prognosis with a median survival of only 4.5 months under traditional salvage therapy with mortality rates reaching 43%, creating an urgent need for new treatment strategies that can eradicate residual disease and prevent relapse [2,3]. More recently developed immunotherapies, including blinatumomab (Blincyto; Amgen Inc., Thousand Oaks, CA, USA) and inotuzumab ozogamicin (Besponsa; Pfizer Inc., New York, NY, USA), have improved outcomes, but the overall prognosis remains only 7-8 months in relapsed patients, with a long-term survival rate remaining below 40% [4]. Such limitations further demonstrate the need for therapies that destroy malignant B cells with greater selectivity and reduced tumor recurrence.

Oncolytic viruses are one such option. They preferentially infect cancer cells and replicate to induce cytolysis and trigger anti-tumor immunity [5]. Unlike chemotherapy, oncolytic viruses amplify their effect with subsequent rounds of replication and can remodel the tumor microenvironment via immunogenic cell death [6]. Adenovirus, herpes simplex virus, and reovirus platforms have shown manageable safety profiles and tumor control with clinical trials conducted in solid tumors [7]. However, hematologic malignancies remain underrepresented, being only a minority of ongoing virotherapy trials [8]. The identification of viral platforms with intrinsic B cell tropism may thus provide a rationale for targeting B-ALL.

Epstein-Barr virus (EBV) is a gammaherpesvirus that causes lifelong latent infection in B lymphocytes and infects over 90% of the human population [9]. Membrane fusion requires sequential receptor interactions: gp350 (glycoprotein 350) binding to complement receptor 2 (CD21) and subsequent engagement of the gH/gL/gp42 (glycoprotein H/glycoprotein L/glycoprotein 42) complex with HLA class II molecules to cause fusion [10]. The gp42 critical function in HLA class II-dependent entry is responsible for the high efficiency of B cell infection [11]. EBV entry into epithelial cells, on the other hand, is gp42 independent but utilizes direct gH/gL binding and fusion to EphA2 (ephrin type-A receptor 2) [12]. Structural studies indicate that gH/gL takes on a four-domain elongated complex with distinct EphA2-binding surfaces [13]. This division of tropism has a critical problem: wild-type EBV replicates productively in epithelial cells but establishes latent infection in B cells [14], meaning it destroys the wrong cell type while failing to kill malignant B lymphocytes [15]

To apply EBV therapeutically, strategies must redirect tropism so that B cells are targeted selectively and epithelial infection is avoided. Mutagenesis studies show that disruption of EphA2 contact residues may block epithelial cell entry, but that preservation of gp42 binding is important to allow B cell infection [16, 17]. Although it is not the focus of this paper, a therapeutic oncolytic EBV would further require reengineering to constantly be in the lytic phase, as otherwise the EBV would remain latent in B lymphocytes.

New computational design tools allow for the reengineering of the gH/gL complex without its tropism to EphA2. Predictive modeling platforms such as AlphaFold3 (DeepMind Technologies, London, UK) can generate high-accuracy structural models of mutant gH/gL complexes with both gp42-HLA (human leukocyte antigen)-DR isotype and EphA2 [18]. Docking algorithms such as HADDOCK (Bonvin Lab, Utrecht University, Netherlands) allow us to create initial docked complexes, and we can take those docked complexes and simulate binding behavior under physiological conditions with molecular dynamics softwares like OpenMM (Simbios Center, Stanford University, CA, USA). Specifically, OpenMM allows us to predict whether our mutations selectively disrupt EphA2 binding while preserving gp42-HLA interactions [19]. This computational pipeline provides an initial evaluation of gH/gL mutations* *and, moreover, of EBV's potential as an oncolytic for B-ALL malignancies before in vitro and in vivo validation[20].

Based on this reasoning, we predict that EBV can be genetically modified as an oncolytic therapy of B-ALL through the introduction of precise mutations in gH/gL that abolish EphA2 binding without impairing gp42-HLA class II interactions. In this work, we create a computational pipeline that creates a mutated variant of gH/gL-gp42 with reduced EphA2 tropism. This study, therefore, provides a foundation for future experimental creation of EBV-based oncolytic virotherapy for B-ALL.

## Materials and methods

Protein structures were obtained from the Protein Data Bank (Worldwide Protein Data Bank, Research Collaboratory for Structural Bioinformatics Protein Data Bank (RCSB PDB), Rutgers University, Piscataway, NJ, USA): EBV glycoproteins gH/gL (PDB: 3PHF, 5T1D) and gp42 (PDB: 3FVC) were retrieved as viral entry proteins, HLA-DR1 (an HLA class II molecule) as the B cell receptor (PDB: 1DLH), and EphA2 as the epithelial cell receptor (PDB: 5WO3). The structures were prepared for simulation by removing water molecules, ligands, and alternate conformations. Also, protonation states were assigned to pH 7.4, and missing atoms were rebuilt. Energy minimization was performed with the CHARMM36m (University of Maryland, College Park, MD, USA) force field to generate stable starting structures [21].

The gH/gL-EphA2 and gp42-HLA-DR interfaces were observed in UCSF Chimera (University of California San Francisco, CA, USA). We used the "Find Clashes/Contacts" tool with a 4.0 Å cutoff to generate contact maps of the two complexes. We found that the majority of direct binding interactions occurred in N-terminal 0-50 residue regions of gH/gL and EphA2, and similarly between gp42 and the 20-45 segment of the HLA-DR β-chain. These regions were the most common amongst the chosen residues as they formed many types of observed bonds between the complexes, including hydrogen bonds, salt bridges, and hydrophobic interactions [22].

To quantify the energetic importance of residues in these pockets, the complexes were submitted to PDBePISA (European Bioinformatics Institute, Hinxton, Cambridge, UK) [23]. For each residue, PDBePISA reported its buried surface area (BSA) and the solvation free energy gain (ΔiG) upon complex formation. High BSA values indicated large contributions to interface burial, while negative ΔiG values indicated favorable stabilizing interactions. Several residues in the 0-50 range met these criteria. In gH/gL, Lys32 (BSA 112 Å², ΔiG -0.55), Arg38 (BSA 96 Å², ΔiG -0.72), Tyr44 (BSA 82 Å², ΔiG -0.40), and Asp47 (BSA 78 Å², ΔiG -0.63) were identified as major contributors. In EphA2, Glu25 (BSA 105 Å², ΔiG -0.61) and Lys29 (BSA 87 Å², ΔiG -0.52) formed complementary stabilizing contacts. For gp42-HLA-DR, key contacts were observed for gp42 residues Ser15 (BSA 74 Å², ΔiG -0.38) and Asp22 (BSA 91 Å², ΔiG -0.69) interacting with HLA-DR β-chain Lys39 (BSA 83 Å², ΔiG -0.41).

These observations were used to engineer disruptive mutations to destabilize EphA2 binding without disturbing gp42-HLA interactions. Positively charged amino acids (e.g., Arg38, Lys32) were substituted with glutamate (negatively charged) to introduce electrostatic repulsion with their complementary EphA2 amino acids (negative). In the same vein, Asp47 (negative) was replaced by lysine (positive) to introduce even more electrostatic repulsion. Polar or hydrogen-bonding residues such as Tyr44 and Ser15 were substituted with phenylalanine, a hydrophobic amino acid lacking a hydroxyl group, to abolish hydrogen-bonding ability. Mutant structures were constructed in UCSF Chimera and relaxed using Rosetta FastRelax (University of Washington, Seattle, WA, USA) to refine side-chain packing [24].

Predicted effects of mutations were evaluated with FoldX PositionScan (Institute for Research in Biomedicine, Barcelona, Spain) and the Rosetta ddG (Gibbs free energy difference) mutagenesis protocol. Both algorithms calculated ΔΔG (change in change of Gibbs free energy) for EphA2 and gp42-HLA complexes. Mutant complexes were selected when they destabilized EphA2 reliably by ≥+1.5≥+1.5 kcal/mol with less than significant impact on gp42-HLA binding (ΔΔG≤+0.5ΔΔG≤+0.5 kcal/mol) [25].

Wild-type and some mutant complexes were also predicted with AlphaFold-Multimer to produce complete mutant structures. Docking refinements were carried out using HADDOCK 2.4, with interface residues being designated from Chimera and PDBePISA. 1000 rigid-body poses were generated by docking, and then 200 rounds of flexible docking and explicit solvent refinement were applied. The top cluster was chosen according to HADDOCK score and interface area.

Molecular dynamics of 20 ns were carried out in OpenMM accelerated using the Compute Unified Device Architecture (CUDA)[26]. Each complex was solvated into a 4-point Optimal Point Charge (water model) water box with 12 Å padding, neutralized, and augmented with 150 mM NaCl. The systems were heated up to 310 K following minimization and equilibrated at 1 atm before 20 ns production runs. Trajectories were collected at 2 fs timesteps. Wild-type and mutant complexes of gH/gL-EphA2 and gH/gL-gp42-HLA-DR were simulated.

Trajectory analysis was performed with MDAnalysis (University of California San Diego, La Jolla, CA, USA) [27]. Root-mean-square deviation of backbone atoms was measured to track global structural stability and interface drift over the course of the simulation. Root mean square fluctuation was computed per residue to illustrate flexible regions of each complex, with flexibility cut-offs at 2.0 Å.

**Figure 1:**
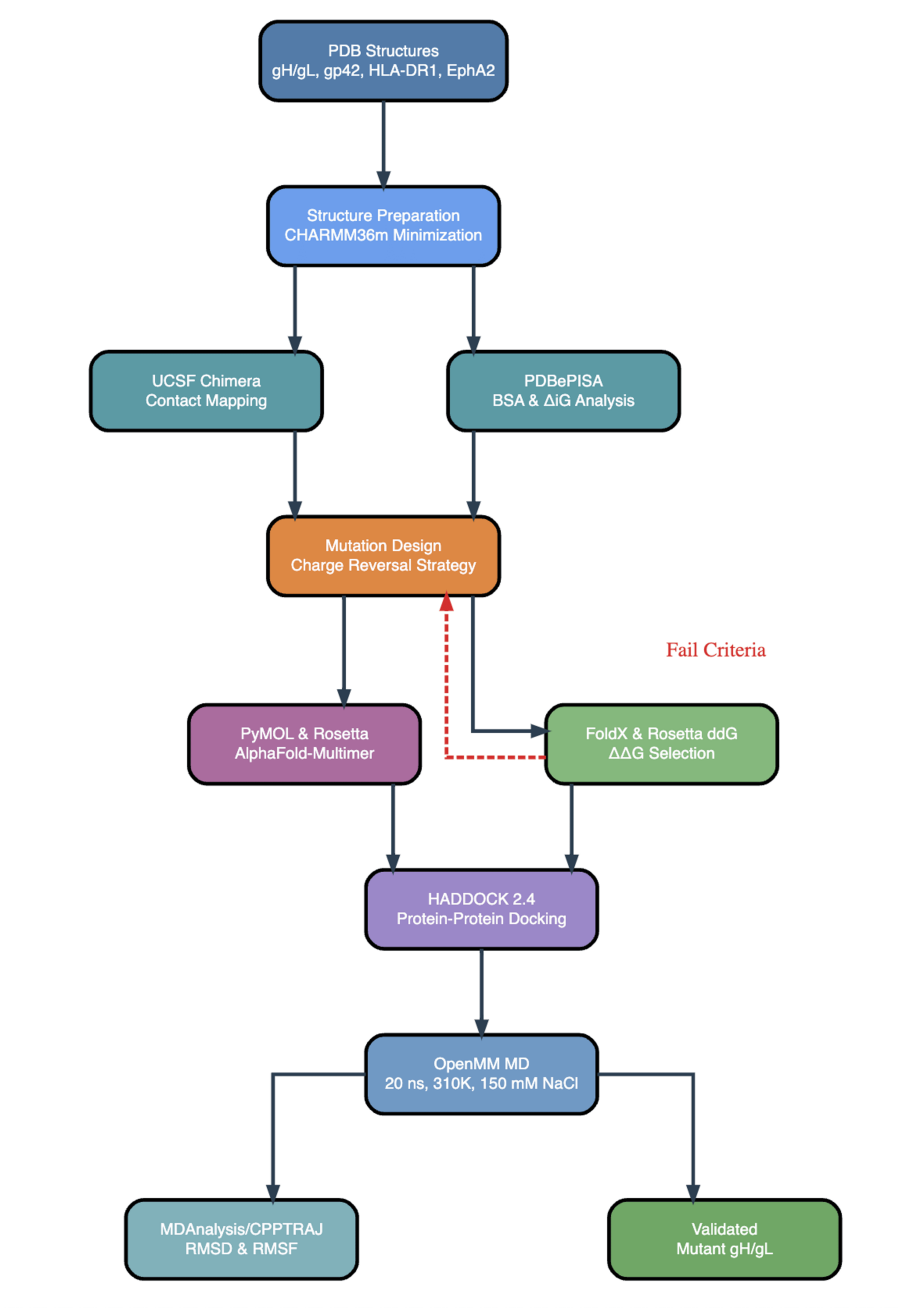
Complete Computational Pipeline of gH/gL mutagenesis and MD Validation RMSD: root mean square deviation; RMSF: root mean square fluctuation; ddG: Gibbs free energy difference; CPPTRAJ:  a trajectory analysis tool from the AMBER molecular dynamics package; ΔiG: solvation free energy gain; PDB: protein data bank; MD: molecular dynamics; ns: nanosecond; K: Kelvin; mM: millimolar; ΔΔG: change in change of Gibbs free energy (difference in binding free energy between wild-type and mutant); gH/gL: glycoprotein H/glycoprotein L; gp42: glycoprotein 42; HLA-DR1: human leukocyte antigen - DR isotype 1; NaCl: sodium chloride; EphA2: ephrin type-A receptor 2; BSA: buried surface area; PyMOL: a molecular visualization system; UCSF: University of California San Francisco; Rosetta (University of Washington, Seattle, WA, USA); AlphaFold-Multimer (DeepMind Technologies, London, UK); FoldX  (Institute for Research in Biomedicine, Barcelona, Spain); HADDOCK 2.4 (Bonvin Lab, Utrecht University, Netherlands); OpenMM (Simbios Center, Stanford University, CA, USA);  MDAnalysis (University of California San Diego, La Jolla, CA, USA); CHARMM36m (University of Maryland, College Park, MD, USA); Chimera (University of California San Francisco, CA, USA); PDBePISA (European Bioinformatics Institute, Hinxton, Cambridge, UK)

The complete computational pipeline is shown in Figure 1. All figures were created on matlibplot [28]. 

## Results

**Figure 2:**
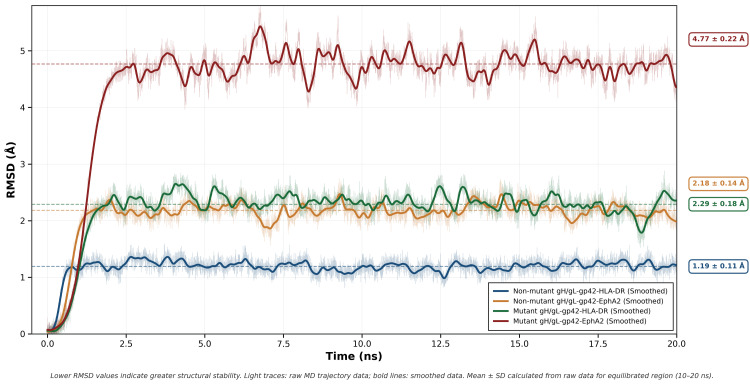
Root mean square deviation (RMSD) of gH/gL–gp42–HLA-DR and gH/gL-gp42–EphA2 complexes over 20 ns This graph compares the structural stability of four protein complexes over a 20-nanosecond molecular dynamics simulation by tracking their RMSD from their starting structures. Lower RMSD values indicate greater structural stability. gH/gL: glycoprotein H/glycoprotein L; gp42: glycoprotein 42; HLA-DR: human leukocyte antigen - DR isotype; EphA2: ephrin type-A receptor 2

The RMSD trajectories of all four complexes are shown in Figure 2.

Non-mutant gH/gL-gp42-HLA-DR complex was low in RMSD throughout the trajectory, with mean deviations of 1.19 ± 0.11 Å. Oscillations were tightly within a band, indicating little conformational change. The mutant gH/gL-gp42-HLA-DR complex was higher in mean deviations of 2.29 ± 0.18 Å and with a greater frequency of oscillations, indicating greater atomic displacement from the starting structure.

The wild-type gH/gL-EphA2 complex exhibited lesser stability overall. RMSD values reached 2.29 ± 0.18 Å and stabilized with moderate fluctuations over the simulation window. The mutant gH/gL-gp42-EphA2 complex showed the largest deviations compared to all others. RMSD increased very quickly in the beginning nanoseconds and reached mean values of 4.77 ± 0.22 Å with larger oscillations, showing heavy instability in the binding conformation.

Through both bound complexes, the non-mutant complexes possessed smaller ranges of RMSD compared to mutants, although the mutant gH/gL-gp42-HLA-DR system was more structurally stable than the mutant gH/gL-gp42-EpAa2 complex (2.29 ± 0.18 Å vs. 4.77 ± 0.22 Å).

**Figure 3:**
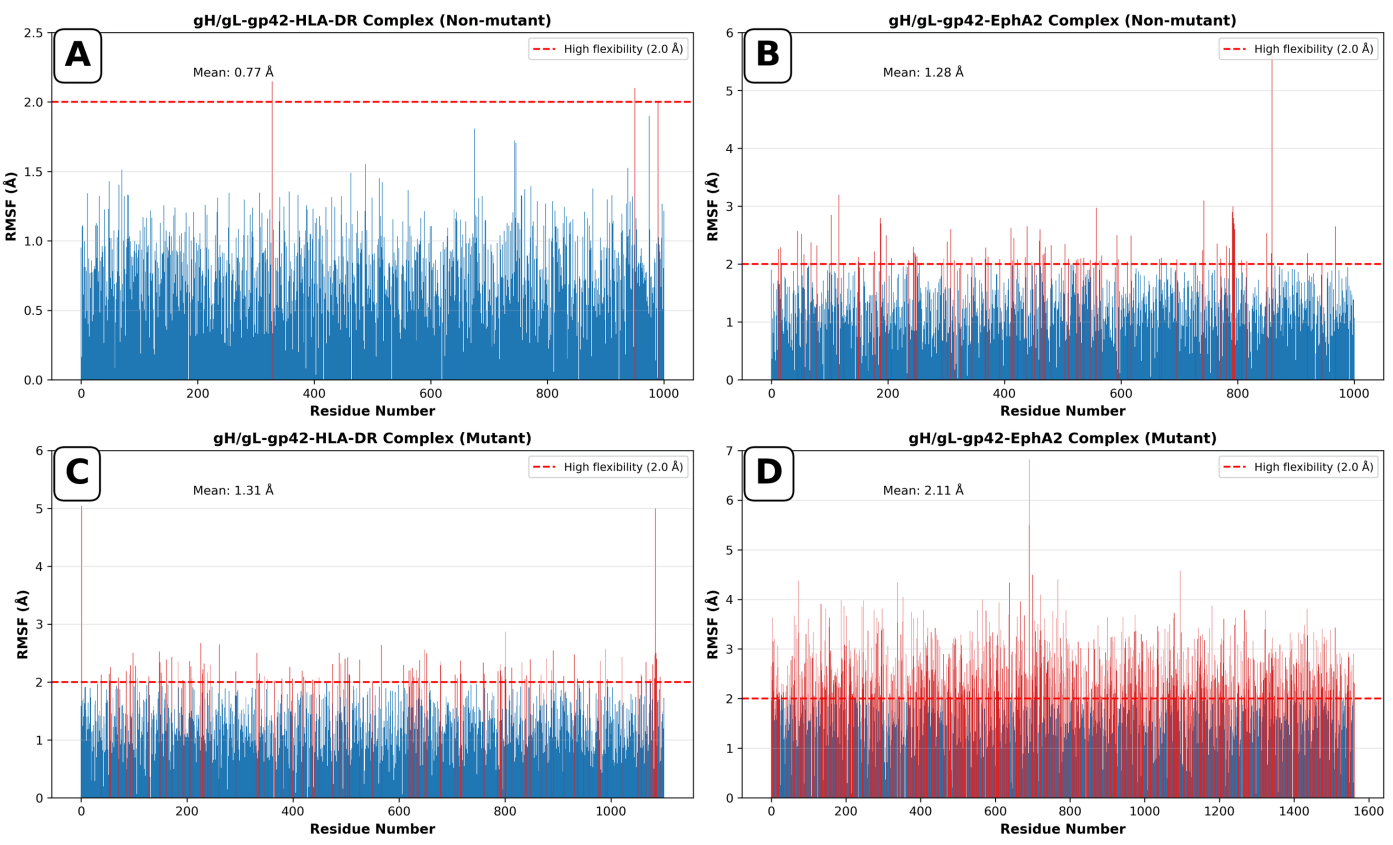
Root mean square fluctuation (RMSF) of gH/gL–gp42–HLA-DR and gH/gL–EphA2 complexes Panel A shows non-mutant gH/gL-gp42-HLA-DR with a mean RMSF of 0.77 Å; Panel B shows non-mutant gH/gL-EphA2 with a mean RMSF of 1.28 Å; Panel C shows mutant gH/gL-gp42-HLA-DR with a mean RMSF of 1.31 Å; and Panel D shows mutant gH/gL-EphA2 with a mean RMSF of 2.11 Å. Panels A and B show non-mutant complexes with low to moderate flexibility (mean 0.77 Å and 1.28 Å, respectively), indicating stable receptor interactions. Panels C and D reveal that the mutations preserved gp42-HLA-DR stability (mean 1.31 Å) while destabilizing EphA2 binding (mean 2.11 Å with 46% of residues exceeding the 2.0 Å threshold). gH/gL: glycoprotein H/glycoprotein L; gp42: glycoprotein 42; HLA-DR: human leukocyte antigen - DR isotype; EphA2: ephrin type-A receptor 2

Figure 3 shows RMSF distributions for all complexes.

In the non-mutant gH/gL-gp42-HLA-DR complex, residues showed low flexibility with an average RMSF of ~0.77 Å and isolated peaks at loop regions. The mutant, however, exhibited more distributed patterns and greater flexibility in several regions with an average RMSF of ~1.31 Å and peaks at ~ 5.0 Å. Different residues at the N-terminal and loop regions showed sustained mobility greater than the threshold value of 2.0 Å.

The non-mutant gH/gL-EphA2 complex had a mean RMSF of ~1.3 Å, with about 12% of residues greater than the 2.0 Å threshold. Peaks were predominantly confined to surface-exposed loops. Substantially greater mobility in the mutant gH/gL-EphA2 complex was seen, with a mean RMSF of ~2.1 Å and nearly half (46%) of residues crossing the 2.0 Å threshold. Maximum flexibility of 6.8 Å was observed at residue 691, indicating a highly mobile region.

Overall, RMSF analysis revealed that non-mutant complexes were rather inflexible with only local fluctuations, while mutant complexes were characterized by more extensive mobility and greater-amplitude peaks, especially in the gH/gL-EphA2 system.

## Discussion

The results show a difference in stability in gH/gLgp42-HLA-DR and gH/gLgp42-EphA2 complexes upon mutagenesis. In the gH/gLgp42-HLA-DR complex, non-mutant RMSD averaged 1.19 ± 0.11 Å while mutant RMSD increased to 2.29 ± 0.18 Å. A less than 3.0 Å deviation indicates that the geometry of binding is still intact. RMSF increased from 0.77 Å to 1.31 Å, with fluctuation being confined to loop parts but not across the interface. These measurements reflect that the gp42-HLA-DR complex is structurally robust in the mutant form [16].

In contrast, the gH/gLgp42-EphA2 complex became destabilized. Non-mutant RMSD was 2.29 ± 0.18 Å on average, while the mutant RMSD increased to 4.77 ± 0.22 Å, well above the stability threshold of single protein-protein contacts. RMSF values grew from 1.28 Å to 2.11 Å, with nearly half the interface residues more than 2.0 Å.

These results agree with the initial hypothesis. gH/gL mutations were targeted to residues within the N-terminal domain involved in EphA2 recognition [16]. Removal of stable contacts within this region disrupts EphA2 binding without blocking gp42 anchoring to gH/gL. This effect is concluded from the RMSD and RMSF analysis. The inference is that B cell entry through gH/gLgp42-HLA-DR is preserved while epithelial entry through EphA2 is blocked [13,18].

The biological explanation is consistent with structural studies of EBV entry. gp42 binds to gH/gL and contacts HLA-DR, with binding surfaces distributed across the two proteins. The multi-contact interface tolerates modest RMSD increases while function is preserved. EphA2 binding depends on a few residues in gH domain II. Mutations there cause instability, as reflected in RMSD greater than 4 Å, which correlates with loss of stable binding [17].

The limitations of this study are clear. Molecular dynamics trajectories were cut off at 20 ns, potentially failing to capture long-timescale rearrangements. Simulations did not include glycosylation, which is known to regulate EBV entry as well as receptor display. The membrane environment was not simulated, where gH/gL operates in a fusion interface within cells [17]. Binding free energies were not calculated; RMSD and RMSF provided stability metrics but not thermodynamic favorability, which were not found. Genetic modification of EBV glycoproteins was not carried out, and therefore, the biological viability of the introduction and maintenance of the mutations is unknown. Additionally, although we hypothesized that a function oncolytic would need to constantly be in the lytic phase, no work directly on the EBV genome was done computationally or otherwise. Additionally, no in vitro or in vivo work was performed, which significantly limits the impact and scope of the study, only acting as a framework or a potential proof of concept.

For clinical translation, additional delivery mechanisms have to be considered. An EBV strain restricted to B cells would have to involve steps to prevent uncontrolled replication [8]. One strategy would be to build conditional replication under tumor-specific promoters, limiting viral spread to the cancer cells [29]. Alternatively, using mutant EBV with suicide gene cassettes would allow for external regulation of replication [29]. EBV entry inhibitors or decoy receptors can be delivered more effectively as proteins or mRNA with the use of lipid nanoparticles [30].

Future work can do the following. First, mutant gH/gL must be expressed in mammalian cells, and binding to EphA2 and HLA-DR quantitated by surface plasmon resonance (SPR) or biolayer interferometry (BLI) [13]. Second, pseudotyped virus particles that carry mutant gH/gL must be tested for entry into B cell and epithelial lines [13]. Loss of epithelial entry but maintenance of B cell entry would confirm the computational prediction. Third, X-ray crystallography or cryo-EM of the mutant complexes should be performed to directly observe structural changes in receptor interfaces [12]. Fourth, extended molecular dynamics simulations with glycosylation and lipid bilayers should be carried out to probe stability under near-physiologic conditions [23]. Change in Gibbs Free Energy can be used to quantify binding energy differences for wild-type and mutant interfaces.

Apart from structural validation, functional assays have to be performed. Engineered EBV viruses carrying the planned mutations have to be assessed in cell culture for selective replication in B cells. Lytic activation assays would ascertain whether the mutant virus replicates and kills malignant B cells but spares epithelial cells. B-cell malignancy animal models would then assess selective oncolysis and in vivo safety.

In brief, the computational findings validate that gH/gL mutations reduce EphA2 stability with no impact on gp42-HLA-DR binding. This lays the foundation for EBV tropism redirection to B cells with minimal entry into epithelial cells.

## Conclusions

The study was an attempt to lay the groundwork for a B-ALL-targeting oncolytic virotherapy using computational analysis. Specifically, it showed that selective mutations to the gH/gL complex in EBV to remove tropism to epithelial cells as a first step to this goal were somewhat successful, wherein molecular dynamics revealed that gp42-HLA-DR interactions remained stable at a mean RMSD of 2.29 ± 0.18 Å while gH/gL-EphA2 interactions destabilized to 4.77 ± 0.22 Å. Limitations include the sole use of in silico models, short trajectory lengths, lack of glycosylation, and overall membrane context. Biochemical assays of receptor binding, pseudovirus entry experiments, structural analysis of mutant complexes, and genetic engineering of replication-competent EBV strains with safety controls should be incorporated in future work for proper in vitro validation. If the therapy can be developed in the future, it may be able to provide a more affordable alternative to therapies such as chimeric antigen receptor T cell (CAR-T). Moreover, oncolytic viruses hold significant clinical value as supplemental therapies due to their potential to stimulate the patient's immune system, a quality also seen in existing oncolytic therapies like talimogene laherparepvec (T-VEC). Furthermore, due to the nature of the vector's high specificity as an oncolytic, it can be used along with radiation or chemotherapies to increase malignant cell destruction for any remaining cells.
